# Swedish emergency hospital surgical surge capacity to mass casualty incidents

**DOI:** 10.1186/s13049-020-0701-8

**Published:** 2020-02-12

**Authors:** Magnus Blimark, Per Örtenwall, Hans Lönroth, Peter Mattsson, Kenneth D. Boffard, Yohan Robinson

**Affiliations:** 1 Institute of Clinical Sciences, Sahlgrenska Academy, Gothenburg University, 413 45 Gothenburg, Sweden; 2 Swedish Armed Forces Centre for Defence Medicine, Gothenburg, Sweden; 3 Department of Military Studies, Swedish Defence University, Stockholm, Sweden

## Abstract

**Background:**

In Sweden the surgical surge capacity for mass casualty incidents (MCI) is managed by county councils within their dedicated budget. It is unclear whether healthcare budget constraints have affected the regional MCI preparedness. This study was designed to investigate the current surgical MCI preparedness at Swedish emergency hospitals.

**Methods:**

Surveys were distributed in 2015 to department heads of intensive care units (ICU) and surgery at 54 Swedish emergency hospitals. The survey contained quantitative measures as the number of (1) surgical trauma teams in hospital and available after activating the disaster plan, (2) surgical theatres suitable for multi-trauma care, and (3) surgical ICU beds. The survey was also distributed to the Armed Forces Centre for Defence Medicine.

**Results:**

53 hospitals responded to the survey (98%). Included were 10 university hospitals (19%), 42 county hospitals (79%), and 1 private hospital (2%). Within 8 h the surgical capacity could be increased from 105 to 399 surgical teams, while 433 surgical theatres and 480 ICU beds were made available. The surgical surge capacity differed between university hospitals and county hospitals, and regional differences were identified regarding the availability of surgical theatres and ICU beds.

**Conclusions:**

The MCI preparedness of Swedish emergency care hospitals needs further attention. To improve Swedish surgical MCI preparedness a national strategy for trauma care in disaster management is necessary.

## Introduction

The Swedish total-defence system, the intimate national civil-military collaboration during the cold war era was able to mobilize all available resources to defend the country [1, 2]. Recently, it has been indicated that Swedish surgical mass casualty incident (MCI) preparedness has decreased substantially since the cessation of the cold war [3, 4]. At the same time the Swedish Defence Commission points out that the security of Sweden’s neighbourhood has declined and that the current threat assessment cannot exclude military measures against Sweden [5].

The Swedish defence healthcare system strongly depends on the support of civilian healthcare during increased levels of mobilisation [6]. Swedish legislation requires public healthcare institutions to maintain MCI preparedness including wartime scenarios [7, 8]. Due to chronic budgetary deficits in most emergency hospitals, investments in the maintenance of MCI preparedness may have received low priority. Until now it is unclear how healthcare budget constraints have affected the regional MCI preparedness [9, 10]. The aim of this paper was to study surgical MCI preparedness in Swedish emergency care hospitals.

## Methods

### Study design

This is a cross-sectional study of Swedish emergency hospital’s surgical capacity related to MCI. It is based on the results of a written questionnaire sent to all hospitals in Sweden with an emergency department. Informed consent was obtained from the respondents in each hospital. No personal data was collected, but hospital resource and infrastructure information, as well as publicly available population data.

### Setting

Sweden has a tax-funded public healthcare system, regulated by the Health and Medical Service Act. The role of the central government is to establish principles and guidelines ensuring that everyone has equal access to healthcare services and to set the political agenda for health and medical care [11]. Sweden is divided in 21 county councils, each given the responsibility to organize and deliver medical care within their geographical area. According to legislation they are also responsible to plan for mass casualty incidents (MCI), which should be based on risk and vulnerability analyses. Apart from the usual hazards related to transports, fires, chemical incidents etc., acts of terror as well as consequences of sudden climate changes and breakdown of critical infrastructure have emerged as potential causes of MCIs. Every emergency hospital is required to have a MCI/“disaster” plan, which can be activated to different levels of alert [10].. The level of alert can be one of the following:

1. Green Alert, (“Stand by”), where a hospital command group keeps itself informed about the situation, takes necessary measures and monitor the development
2. Yellow Alert (“Partial mobilization”), where a number of pre-defined clinical key functions of the hospital are activated to receive casualties
3. Red Alert (“Full mobilization”) where all available staff within emergency and supporting disciplines are alerted

### Questionnaire

The number of surgical teams, number of ICU beds and surgical theatres are, according to the reference group, well-established numerical key performance indicators for surgical capacity and are commonly used benchmarks in surgical trauma centres. The reference group consisted of disaster preparedness representatives from the National Board of Health and Welfare, the Civil Contingencies Agency, and the Armed Forces Centre for Defence Medicine.

The survey was sent by The National Board of Health and Welfare directly to all Swedish emergency hospitals and the military field hospital as part of a larger survey of Swedish trauma care.

The survey was addressed to the Heads of the Departments of surgery and the Departments of anaesthesiology/ICU. These were contacted by telephone; the purpose of the study was explained, and they were requested to either answer the questionnaire, which was sent by e-mail to the respondents.

The questionnaire form included an introductory text on the background of the trauma investigation and information on how data was going to be presented. A translated version of the questions is presented in Table 1.

**Table 1:** Translation of the surgical preparedness survey

Question #	Question	Answer format
1a	How many surgical teams can your hospital mobilize within 0.5 h?	(numeric)
1b	How many surgical teams can your hospital mobilize within 2 h?	(numeric)
1c	How many surgical teams can your hospital mobilize within 8 h?	(numeric)
2	Which is the estimated endurance for your hospital regarding the trauma-teams?	(numeric, days)
3	How many theatres, designed and equipped for and immediately available to receive seriously injured trauma-patients, are available?	(numeric)
4	How many ICU-beds, able to treat seriously injured trauma-patients, are available?	(numeric)
5	What length of time is required before your ICU-capacity is available to receive the first trauma-patient?	(numeric, hours)
6	What is the estimated endurance for your increased ICU-capacity?	(numeric, days)
7	How many trauma-patients can be monitored and treated with intermediate-care if there are no possibilities to transfer patients to other hospitals?	(numeric)

### Population data

Swedish population data was obtained by the open national population registry maintained by Statistics Sweden.

### Statistical analysis

Baseline data and distribution are presented in tables. Differences regarding to hospital type and region are presented in tables. Group differences were tested with the Student t-test. As relevant hospital types, university hospitals, county hospitals and private hospitals with emergency departments, were included. Following regions were defined: West (Västra Götalandsregion, Halland), East (Stockholm, Gotland, Uppsala), South (Skåne, Blekinge, Kalmar, Kronoberg), North (Dalarna, Jämtland, Norrbotten, Västerbotten, Västernorrland, Gävleborg), and Central (Östergötland, Sörmland, Västmanland, Örebro, Jönköping).

The data table was processed using Microsoft Excel for Mac (version 16.26).

## Results

### Study population

In total, 54 hospitals received the questionnaire, of which 53 answered all questions (98%). Included were 10 university hospitals (19%), 42 county hospitals (79%), and 1 private hospital (2%).

### Capacity after full mobilization (“red alert”)

#### Surgical theatres

The number of surgical theatres equipped to deal with major trauma in Sweden was 433. University hospitals contributed 36% of the total number of surgical theatres, county hospitals 63% and private hospitals 2% (Table 2).

**Table 2:** Surgical surge capacity related to hospital type

	n	Surgical trauma teams 30 min	Surgical trauma teams 2 h	Surgical trauma teams 8 h	Surgical theatres	ICU beds	hours until full ICU capacity	Intermediate care beds
University hospital	10	27	52	108	155	171	5.4	214
County hospital	42	75	174	286	271	301	1.8	321
Private hospital	1	3	5	5	7	8	N/A	8
All hospitals	53	105	231	399	433	480	2.5	543

#### Intensive care units

The number of ICU beds in Sweden was 480. University hospitals contributed 36%, county hospitals 63% and private hospitals 2% (Table 2).

#### Surgical trauma teams

The number of surgical trauma teams in Sweden could be increased from 105 available teams within 30 min to 399 teams 8 h after activation of a Red Alert. County hospitals contributed with 72% of the available teams, while university hospitals contributed 27% and private hospitals 1% (Table 2).

### Surgical surge dynamics

**Fig. 1:**
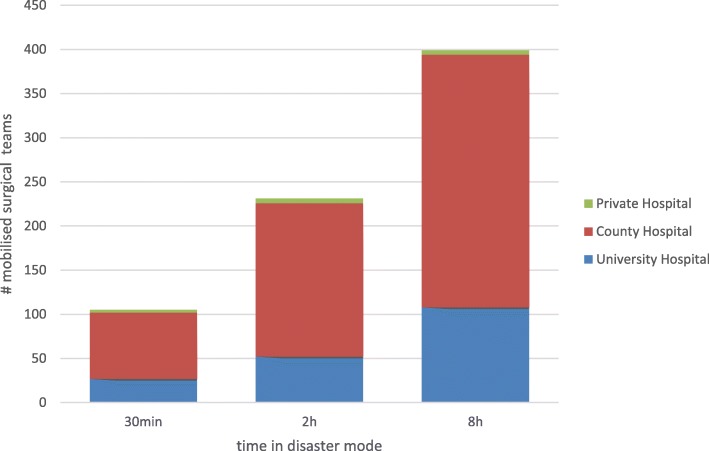
The contribution to the surgical team surge capacity was related to hospital type

The mobilised surgical teams increased from 105 teams after 30 min to 231 teams after 2 h and to 399 teams after 8 h (Fig. 1). The time to mobilize the full capacity of 480 ICU beds in Sweden (mean 2.5 h) was longer in university hospitals (5.4 h) as compared to county hospitals (1.8 h) (*p* < 0.001) (Table 2).

### Regional differences

There were regional differences in the availability of operation theatres, surgical teams, and ICU-beds. These are presented in Table 3.

**Table 3:** Regional differences with regards to population density, number of hospitals, and surgical capacity

*Region (University cities)*	*Hospitals*	*Population*	*Area* *km*^*2*^	*Population density per km*^*2*^	*Surgical teams after 8 h*	*Surgical theatres*	*ICU beds*	*Surgical teams after 8 h per 100 k*	*Surgical theatres per 100 k*	*ICU beds per 100 k*
*West (Gothenburg)*	9	2,015,607	29,227	68.96	65	67	106	3.22	3.32	5.26
*East (Stockholm, Uppsala)*	10	2,696,566	17,844	151.12	106	113	113	3.93	4.19	4.19
*South (Malmö, Lund)*	10	1,944,315	33,691	57.71	70	101	93	3.60	5.19	4.78
*North (Umeå)*	10	1,466,824	268,911	5.45	64	76	82	4.36	5.18	5.59
*Central (Linköping, Örebro)*	14	1,667,757	40,171	41.52	94	76	86	5.64	4.56	5.16
*All regions*	*53*	*9,791,069*	*389,844*	*25.12*	*399*	*433*	*480*	*4.08*	*4.42*	*4.90*
*Military field hospital*	1	14,600	N/A	N/A	6	6	12	41.10	41.10	82.19

In the East the number of ICU beds do not match the number of surgical teams and operation theatres, in contrast to the West (Gothenburg area), where the number of ICU beds that can be mobilized are approximately double the number of surgical teams and surgical theatres.

### Contribution of military surgical capacity

The military surgical capacity is dimensioned for an armed force with 14,600 soldiers. Thus, the field hospital has 6 surgical teams, 6 operation theatres and 6 ICU beds (Table 3). Compared to civilian care the military field hospital represents 1.5% of the national surgical teams, 1.4% of the national surgical theatres and 2.5% of the national ICU bed capacity.

## Discussion

This study presents an assessment of the emergency hospitals’ surgical disaster preparedness from a nationwide Swedish perspective. We found regional differences regarding availability of ICU-beds, surgical theatres, trauma teams, and differences between university hospitals, county hospitals and private hospitals in surgical surge capacity.

### Swedish surgical disaster preparedness

The preparedness of Swedish hospitals for disaster, crisis or homeland defence gradually diminished after the cessation of the cold war. The military surgical capacity was dramatically reduced in 2002. Similarly, the civilian homeland defence resources were down prioritized. In 2015 Sweden had a total defence healthcare system with reduced resources for surgical MCI preparedness [4].

In total, the combined surgical capacity was 399 surgical teams, 433 surgical theatres and 480 ICU beds (Table 2). The possible military contribution to these numbers was 6 surgical teams, 6 operation theatres and 12 ICU beds. These figures depend on a fully operational hospital infrastructure, with unaltered availability of hospital staff, pharmaceuticals, medical supplies and blood products. In the situation of a national emergency, these conditions cannot be taken for granted.

With 2.4 beds per 1000 inhabitants, Sweden had one of the lowest numbers of hospital beds per capita in 2015 according to a report from the Organisation for Economic Co-operation and Development (OECD) [12]. This came at the cost of overutilization of hospital beds. The Swedish Association of Local Authorities and Regions reported an overutilization with 5.4 per 100 hospital beds [13]. Therefore, the system struggles with the preparedness for a large number of patients in case of an MCI.

The capability to receive, treat and care for patients arriving after a large-scale MCI has not been analysed in depth in this study, but it is likely to be affected negatively by:

- Insufficient prehospital transport capacity both by ground-based and aeromedical evacuation
- Technical vulnerability of Swedish hospitals
- Insufficient pharmaceutical and medical supplies
- Under-dimensioned number of hospital-beds and ICU-units
- Insufficient number of trauma-competent staff at Swedish hospitals

### Regional differences in disaster preparedness

The population density distribution of Sweden with a very low population density in the northern regions and a relatively high population density in the South reveals the geographical challenges of MCI preparedness. Even though few people live in the North, mining industry, dense railway and air traffic are possible sources for MCI. It is therefore reasonable that the number of hospital surgical theatres and ICU beds per capita are higher in the northern than in the southern regions (Table 3). There is currently no consensus on the minimally necessary number of surgical theatres and ICU beds related to population density which remains as a subject of further research.

### Importance of study results for Swedish healthcare policy

Most injured in a mass casualty incident in Sweden will be taken care of by the national civilian healthcare system, regardless of the cause. This is also true for military personnel wounded in an armed conflict. Therefore, the national disaster preparedness is directly associated to armed forces health from a total defence perspective. The national resilience, being one of the critical pillars of total defence is associated to the ability of providing an adequate level of medical care [5]. Of course, Sweden is not the only country struggling with hospital disaster preparedness [14–16]. Thus, multinational coordination of disaster preparedness efforts as for instance the European Commission Disaster Risk Management Knowledge Centre will improve interoperability and mitigate the risk of repeating mistakes [17].

Sweden’s defence policy is relying on a strong total defence, and partner nations’ armies joining Swedish forces against foreign hostilities. As a trustworthy host and partner nation, Sweden needs to be prepared to provide host nation medical support to friendly military troops [18].

### Strengths and limitations

Survey studies are typically prone to errors related to coverage, sampling, measurement, and nonresponse [19]. This study eliminated coverage errors by including all emergency hospitals in Sweden. The population of interest was clearly identified, and due to the nationwide design sampling was not necessary. All but one emergency hospital responded, thus the nonresponse rate was less than 2%. Possible reasons for the high response-rate were a sense of importance of this survey as well as the involvement of the Swedish National Board of Health and Welfare. The questions in the survey were directly related to the topic of interest - surgical surge capacity. Similar questions have been successfully used in previous studies [4].

The survey has identified the dynamics of mobilizing certain key assets but not the dynamics of patient flow. Instead of focusing on isolated parameters, a more relevant question could have been to ask the hospitals to describe the number of major trauma patients they can manage within certain timeframes. However, as the experience among Swedish hospitals to deal with MCI is limited, the expected accuracy of such data remains questionable. For similar reasons questions regarding hospital endurance, as a key performance indicator for disaster preparedness, was not included in this survey [20].

Most hospitals train their trauma-teams at their emergency department with exercises including a limited number of simulated casualties. Very few hospitals train for MCI, focusing on the hospital’s entire ability to deal with a very large number of trauma casualties. Unfortunately, the disaster preparedness plans of most hospitals are rarely tested in large scale exercises.

Beyond that, the study focuses merely on hospital surgical capacity. It did not include prehospital capacity, nor inter-hospital patient transfer capacity. The ability of the surgical capacity of neighbouring countries to assist has not been subject of this survey.

### Future areas of research

While conducting this study the following knowledge gaps have been identified that need to be investigated in order to create a better understanding of the national disaster preparedness in Sweden as well as to identify solutions to increase that readiness:

- Prehospital capacity and inter hospital transfer capacity.
- Benchmarking of countries’ disaster preparedness.
- Swedish hospitals capacity to deal with large number of casualties, identifying the bottle necks of the organization.
- The effect of disaster exercises on the same hospitals, with figurants, comparing the outcome with simulations including identical parameters
- Access to critical pharmaceutical products and medical disposables
- Endurance of Swedish hospitals regarding availability to sustain in disaster-mode.
- Civilian-military collaboration in crisis management.

## Conclusion

This study was an attempt to quantify current Swedish national surgical preparedness in response to MCI. Our results must be followed by recurrent nationwide surveys to identify early trends in hospital preparedness. Sweden is facing the demanding challenge to increase its national surgical surge capacity. Swedish surgical MCI preparedness needs a national long-term strategy for trauma system development in MCI based on reliable key performance indicators is necessary.

## Data Availability

The datasets used and/or analysed during the current study are available from the corresponding author on reasonable request.

## Abbreviations

ICU: Intensive Care Unit
MCI: Mass Casualty Incident
OECD: Organisation for Economic Co-operation and Development
